# How to make full use of dental pulp stem cells: an optimized cell culture method based on explant technology

**DOI:** 10.3389/fbioe.2024.1324049

**Published:** 2024-03-18

**Authors:** You Wu, Jiangling Sun, Wang Wang, Yao Wang, Reinhard E. Friedrich

**Affiliations:** ^1^ Department of Stomatology, Chengdu Seventh People’s Hospital (Affiliated Cancer Hospital of Chengdu Medical College), Chengdu, China; ^2^ The Department of Preventive Dentistry, The Affiliated Stomatological Hospital of Southwest Medical University, Luzhou, China; ^3^ Department of Science and Education, Guiyang Stomatological Hospital, Guiyang, China; ^4^ Department of Oral and Maxillofacial Surgery, University Medical Center Hamburg-Eppendorf, Hamburg, Germany; ^5^ Center for Plastic & Reconstructive Surgery, Department of Stomatology, Zhejiang Provincial People’s Hospital, Affiliated People’s Hospital, Hangzhou Medical College, Hangzhou, China; ^6^ Department of Periodontics, Preventive and Restorative Dentistry, University Medical Center Hamburg-Eppendorf, Hamburg, Germany

**Keywords:** human dental pulp stem cells, tissue engineering, proliferation ability, cellular differentiation, methodology, cell culture, explant technology

## Abstract

**Introduction:**

Dental pulp stem cells from humans possess self-renewal and versatile differentiation abilities. These cells, known as DPSC, are promising for tissue engineering due to their outstanding biological characteristics and ease of access without significant donor site trauma. Existing methods for isolating DPSC mainly include enzyme digestion and explant techniques. Compared with the enzymatic digestion technique, the outgrowth method is less prone to cell damage and loss during the operation, which is essential for DPSC with fewer tissue sources.

**Methods:**

In order to maximize the amount of stem cells harvested while reducing the cost of DPSC culture, the feasibility of the optimized explant technique was evaluated in this experiment. Cell morphology, minimum cell emergence time, the total amount of cells harvested, cell survival, and proliferative and differentiation capacity of DPSC obtained with different numbers of explant attachments (A1-A5) were evaluated.

**Results:**

There was a reduction in the survival rate of the cells in groups A2-A5, and the amount of harvested DPSC decreased in A3-A5 groups, but the DPSC harvested in groups A1-A4 had similar proliferative and differentiation abilities. However, starting from group A5, the survival rate, proliferation and differentiation ability of DPSC decreased significantly, and the adipogenic trend of the cells became more apparent, indicating that the cells had begun to enter the senescence state.

**Discussion:**

The results of our study demonstrated that the DPSC obtained by the optimized explant method up to 4 times had reliable biological properties and is available for tissue engineering.

## 1 Introduction

Tissue engineering aims to replace, repair, or regenerate organs and tissues damaged by trauma, disease, or congenital malformations (Sahakyants and Vacanti, 2020), such as deficiencies in skin, bone, nerves, and tendons (Gomri et al., 2022), by creating living human structures. Due to recent developments in cell biology, biomaterials, and other subject areas, research on tissue engineering has made great strides. For instance, the clinical translation of tissue engineering of complex tissues such as skin, ears, and blood vessels has been successfully performed with good prognostic results (Hibino et al., 2010; Vig et al., 2017; Zhou et al., 2018).

In tissue engineering, suitable stem cells are critical to ensure tissue reconstruction’s effectiveness. Stem cells are a class of cells with high proliferation and differentiation ability, which can play an essential role in tissue and organ reconstruction (Hoang et al., 2022) and are usually divided into 3 types: embryonic stem cells (ESCs), induced pluripotent stem cells (iPSCs) and mesenchymal stromal cells (MSC) (Tong et al., 2015). Although ESCs have the best stemness theoretically, their derivation from human embryos poses various ethical difficulties (Golchin et al., 2021). iPSCs are stem cells that reprogram adult cells back to their original state; nevertheless, cells produced from iPSCs have risk of causing tumor formation because of reprogramming factors involved with tumor development, which has limited their research and clinical applications (Yoshihara et al., 2017). Compared to the other two sources of stem cells, MSC is considered a more desirable candidate than the other two sources of stem cells due to fewer ethical issues and a more comprehensive range of tissue sources, such as fat, bone marrow, cord blood and teeth (Hoang et al., 2022). Among them, dental pulp-derived MSCs are known as dental pulp stem cells (DPSC), first discovered and isolated successfully by Gronthos et al., in 2000 (Gronthos et al., 2000). DPSCs have been reported to have excellent proliferative capacity and multidirectional differentiation potential and can be differentiated into various cell types *in vitro*, including adipocytes, chondrocytes, neuronal cells, and myocytes (Jo et al., 2007; Wei et al., 2007; Chang et al., 2014; Kawashima et al., 2017). Another massive advantage of DPSC is its accessibility: DPSC can be easily separated from extracted teeth, such as obstructed third molars, which are often discarded as medical waste (Pilbauerova et al., 2021). In addition, compared to other sources of MSC, such as bone marrow and fat, the acquisition of DPSC is less traumatic for the donor, which makes them uniquely advantageous for a wide range of medical research and applications.

Ferrúa et al. analyzed more than 200 papers on experiments related to DPSC and found that two main techniques are used to isolate them: exosome and enzymatic (Ferrua et al., 2017a). The most common method is the enzymatic technique, in which hydrolytic enzymes such as collagenase, dispase, and trypsin decompose the extracellular matrix to obtain DPSC (Kerkis et al., 2006; Asgary et al., 2014). The most significant advantage of the enzymatic technique is its time-saving, enabling DPSC isolation in a relatively short period - usually a few hours. However, it also has obvious drawbacks: one is the high cost of using collagenase, which often costs hundreds of dollars for 1g of collagenase by weight; and the other is that the enzymatic method carries certain risks, as the inappropriate type of enzyme, too high enzyme concentration, and too long a digestive time can irreversibly cause damage to the quantity, activity, and function of DPSC, which can hurt the application of stem cells (Huang et al., 2006a). On the other hand, explant technology, although less frequently used in laboratory studies due to the longer time spent on cell culture (usually 1–2 weeks), has the irreplaceable advantages of lower cost of cell isolation and better assurance of cell performance due to relatively lower risk during operation (Huang et al., 2006b).

The most desirable source of stem cells needed in tissue engineering is still considered autologous stem cells, as they do not cause immune rejection and involve fewer ethical issues (Kim et al., 2018; Volarevic et al., 2018). DPSC has promising future applications in tissue engineering due to its multipotent differentiation ability. Although human clinical studies on DPSC are still relatively few due to reasons such as incompletely confirmed safety and cost of treatment, animal experiments on DPSC have been relatively abundant, which amply demonstrates their promising application in tissue engineering. Barone et al. implanted DPSC and cell scaffolds into nude mice and found that the experimental group obtained better *in vivo* vascular network generation (Barone et al., 2023). Yamaguchi et al. applied DPSC intravenously to treat mice in an ischemia-reperfusion myocardial injury model and found that apoptosis and inflammation in cardiomyocytes were significantly suppressed (Yamaguchi et al., 2015). In conclusion, dental pulp stem cells have a wide range of applications and are, therefore, in high demand. However, a potential problem with the application of DPSC is the small pulp tissue size, especially for deciduous teeth, which results in a small number of primary cells available for culture. Consequently, producing stem cells sufficient for clinical use requires multiple passages, which may reduce the potency of cultured cells (Tatullo et al., 2015). Therefore, to ensure the cells’ performance minimizes adverse reactions in recipients, it is generally advocated that stem cells be frozen in advance when the donor is at a young age (Wang et al., 2022). Several cryoprotective agents for MSC cryopreservation have been investigated to reduce the formation of ice crystals inside and outside the cells, thus preventing cell loss and minimizing cell dehydration (Marquez-Curtis et al., 2015; Patel et al., 2023). However, existing cryopreservation techniques still have drawbacks: freeze-thaw stress may cause cytoskeletal damage, leading to the inevitable death of some cells (Oh et al., 2023). Hence, figuring out a method that maximizes the preservation of healthy DPSC is imperative for their possible future applications in tissue engineering and regenerative medicine.

During our culture of DPSC, we stumbled upon the feature that the pulp tissue used for the explant technique can be utilized repeatedly. After the primary cells have grown out and formed sufficient confluence, the original pulp tissue block can be transferred to a new cell culture dish to continue the adherent culture to collect as much DPSC as possible. This method significantly saves the cost of DPSC isolation as it does not require any digestive enzymes. In addition, compared to the enzymatic method, the repeated outgrowth method does not risk causing cell damage and loss during the operation, which maximizes the amount of cells harvested. However, there is a limit to the division of normal somatic cells due to the shortening of telomeres during cell division (Wright and Shay, 2000). Hayflick first described the limited replicative lifespan of normal cells in culture, often called the “Hayflick limit” (Hayflick and Moorhead, 1961). Cells that exceed this limit permanently stop dividing and enter a state of cellular senescence, irreversibly stalling in the G1 phase of the cell cycle (Ohtani et al., 2009). Alraies et al. reported that DPSCs, which were initially highly proliferative, showed a marked decrease in osteogenic and chondrogenic differentiation and an increase in adipogenesis after prolonged expansion (Alraies et al., 2017). Therefore, the purpose of this experiment is to explore the maximum number of operations of the repeated explant technique under the premise of ensuring the cell performance to verify the feasibility of the optimized explant technique as well as to obtain the maximum number of healthy DPSC, which can provide a more theoretical basis for the application of DPSC in tissue engineering.

## 2 Materials and methods

### 2.1 Preparation of samples

At the University Medical Centre Hamburg-Eppendorf, four impacted third molars were extracted between May 2022 and June 2022 from healthy teens between the ages of 15 and 19. The institutional review board of the Hamburg Medical Chamber approved the experimental procedure (IRB-vote # REC 1712/5/2008). The collected teeth were rinsed with 0.7% saline and placed in DPBS solution. The samples were processed within 24 h.

### 2.2 Cell culture

High-speed turbine drills are used to cut through the tooth’s hard tissue and remove the pulp [10]. The pulp tissue was then cut into small pieces around 1 mm^3^ and placed in 24-well plates (6 tissue blocks for each group for start). After they were attached at the bottom, DMEM culture medium (containing 10% FBS and 100 U/mL Pen/Strep) was used to soak the tissue blocks, after which the cells were left to grow. The cell culture plates were then incubated in 5% CO2 at 37°C. When the colony confluence reached an ideal level, the tissue blocks were transferred with sterile forceps to new 24-well plates and cultured as described above. The cells obtained from the first attachment of the tissue block were recorded as group A1, and the cells obtained from subsequent attachment were recorded as groups A2, A3, A4...and so on.

### 2.3 Record of cellular morphology and the shortest emergence time for cells

Two well-trained and experienced experimenters observed each group of cell morphology under the microscope daily and recorded the cells’ earliest appearance. After the colonies reached 80% confluency, cells were isolated by digestion with 0.05% trypsin (Cat. NO. 25300-054, Gibco, Paisley, UK) and passaged.

### 2.4 Flow cytometry

The cultured 3rd generation DPSC were collected for flow cytometry assay. Antibodies against CD34, CD45, CD73, CD90, and CD105 (all PE-coupled antibodies) were selected to detect stem cell surface antigens. After staining with the above antibodies and live-dead stain, cells were washed with PBS and stained with BD LSRFortessa Cell Analyzer (Becton Dickinson Bioscience, Becton, USA) and BD FACSDiva software V6.1 (Becton Dickinson Bioscience, USA) to detect the cells. The obtained data were analyzed using FlowJo software V10.0 (Treestar Inc., Ashland, USA).

### 2.5 Cell survival rate

The cultured 3rd generation DPSC in each group were plated at a density of 4 × 10^4^/mL on TCC (tissue culture coverslips, Sarstedt, Nümbrecht, Germany). Then, they were cultured in the incubator for 24 h. 125 μL propidium iodide at 50 μg/mL and 60 μL fluorescein diacetate at 20 μg/mL were used for the staining. Later, the stained samples were observed using the fluorescence microscope. Cell survival rate = number of (green-stained cells/total cells) × 100%.

### 2.6 Proliferation testing with MTS assay

The fourth generation DPSC were seeded in 96-well plates at a density of 1 × 10^4^/mL, equating to 1 × 10^3^ cells/well and incubated at 37°C in 5% CO2 incubators. The fresh DMEM culture medium was changed every 3 days. The cell viability of each group was tested daily with the MTS kit (Cat. NO. G1111, Promega, USA) according to the instructions. Three parallel wells were set up for each group of samples, and every group was tested for 7 consecutive days. After adding 20 μL of the MTS mix assay solution and reacting for 4 h, the reading at 490 nm was detected and recorded using a microplate reader (Thermo Fisher Scientific, Waltham, MA, USA).

### 2.7 Assessment of differentiation abilities

#### 2.7.1 Osteogenic and adipogenic differentiation

Induction was conducted using fifth-generation DPSC seeded in 6-well plates at 4 × 10^4^ per well. Cells were counted and standardized using an EVE automated cell counter (NanoEntek, Seoul, Korea) before seeding to ensure accurate cell counts. Osteogenic and adipogenic induction was initiated after the cells had grown to 60%–70% density, respectively. For osteogenic differentiation, cells were subjected to a 3-week culture in the osteogenic induction medium (DMEM supplemented with 10% FBS, 10 mmol/L glycerophosphate, 5 mmol/mL ascorbic acid, and 1 mol/L dexamethasone). The culture medium was refreshed at 3-day intervals. Regarding adipogenic differentiation, cells were cultured in adipogenesis induction medium (DMEM containing 10% FBS, 5 μg/mL insulin, 0.5 mmol/L 3‐isobutyl‐1‐methylxanthine, and 10 μmol/L dexamethasone) for 3 weeks, with the medium being replaced every 3 days.

#### 2.7.2 Quantitative analysis of osteogenic differentiation

Stain each group of cells after osteogenic induction with Alizarin Red solution. On the 22nd day, cellular fixation was performed using paraformaldehyde (PFA; Electron Microscopy Sciences, Fort Washington, PA, USA) for 20 min, followed by three consecutive washes with DPBS. Subsequently, the treated cells underwent staining for 10–15 min utilizing a 0.1% Alizarin red S solution (Catalog Number: GT6383, Glentham, Germany). After staining, cells were rinsed 3 times with DPBS, and the inverted microscope was used to observe and take pictures of the stained calcium nodules. After removing the staining solution and rinsing the wells with DPBS solution, 750 μL of 10% glacial acetic acid solution was added to each well to dissolve the stained calcium nodules, followed by 750 μL of 10% ammonium hydroxide to re-form the calcified precipitate solution. The obtained solution was transferred to a 96-well plate at 100 μL per well and read at 405 nm using a microplate reader. The higher the absorbance of the solution, the more calcium nodules are produced by the same amount of DPSC.

#### 2.7.3 Quantitative analysis of adipogenic differentiation

Stain each group of cells after adipogenic induction with Oil Red O solution. On the 22nd day, cell fixation was carried out using paraformaldehyde for 20 min, followed by three washes with DPBS. Subsequently, the stimulated cells underwent staining with a 0.5% Oil Red O solution (Catalog Number: O1391-250ML, Sigma-Aldrich, USA) for 10–15 min. After staining, cells were rinsed 3 times with DPBS, and the stained lipid droplets in the cytoplasm were seen and photographed using an inverted microscope. After removing the staining solution and rinsing the wells with DPBS solution, 1 mL isopropanol solution was added to each well to dissolve the lipid drops. The obtained solution was transferred to a 96-well plate at 100 μL per well and read at 540 nm using a microplate reader. The higher the absorbance of the solution, the more lipid droplets are produced by the same amount of DPSC.

### 2.8 Gene expression

Total RNA in osteogenic and adipogenic groups was extracted using TRIzol reagent (Cat. No. 15596026, Ambion, TX, USA), which was then reverse transcribed using the GoScriptTM RT assay kit. Lipoprotein Lipase (LPL), Peroxisome proliferator-activated receptor-γ (PPAR-γ), alkaline phosphatase (ALP), runt-related transcription factor 2 (RUNX2), type I collagen (COL I) and osteocalcin (OCN) were chosen as specific differentiation genes. GAPDH and Pgk 1 were selected as the reference housekeeping genes. The Cq result is calculated as 2^−ΔΔCT^ after normalization. Primer sequences are in Table 1 below.

**TABLE 1:** Primer sequences of adipogenic and osteogenic-induced gene expression.

Primer	Direction	Sequence	Length of products (bp)
LPL	Forward	ACA​AGA​GAG​AAC​CAG​ACT​CCA​A	76
	Reverse	GCG​GAC​ACT​GGG​TAA​TGC​T	
PPAR-γ	Forward	GGG​ATC​AGC​TCC​GTG​GAT​CT	186
	Reverse	TGC​ACT​TTG​GTA​CTC​TTG​AAG​TT	
ALP	Forward	ACT​GGT​ACT​CAG​ACA​ACG​AGA​T	97
	Reverse	ACG​TCA​ATG​TCC​CTG​ATG​TTA​TG	
RUNX 2	Forward	TGG​TTA​CTG​TCA​TGG​CGG​GTA	97
	Reverse	TCT​CAG​ATC​GTT​GAA​CCT​TGC​TA	
Type I collagen	Forward	GTG​CGA​TGA​CGT​GAT​CTG​TGA	119
	Reverse	CGG​TGG​TTT​CTT​GGT​CGG​T	
Osteocalcin	Forward	GGC​GCT​ACC​TGT​ATC​AAT​GG	110
	Reverse	GTG​GTC​AGC​CAA​CTC​GTC​A	
GAPDH	Forward	GAG​TCA​ACG​GAT​TTG​GTC​GT	185
	Reverse	GAC​AAG​CTT​CCC​GTT​CTC​AG	
Pgk 1	Forward	TGG​ACG​TTA​AAG​GGA​AGC​GG	152
	Reverse	GCT​CAT​AAG​GAC​TAC​CGA​CTT​GG	

### 2.9 Statistical analysis

The data was analyzed by conducting one-way ANOVA and two-way ANOVA to examine the differences in average values among the groups. A significance level of 0.05 was employed. The software packages SPSS 25.0 (SPSS Inc., IL, Chicago, USA) and GraphPad Prism 9.0.0 (GraphPad Software, CA, San Diego, USA) were utilized for the statistical analysis.

## 3 Results

### 3.1 Identification of specific stem cell markers

**FIGURE 1:**
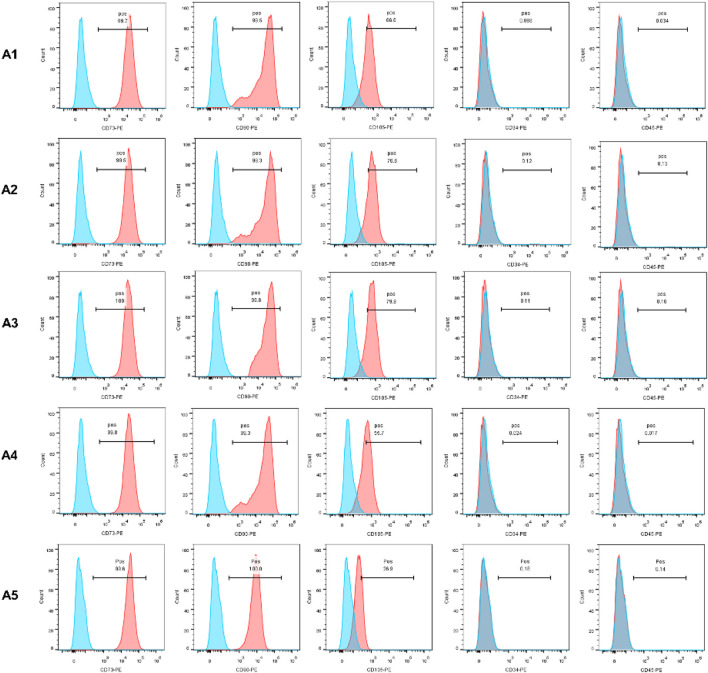
Identification of MSC by flow cytometry. The expression of CD73, CD90 and CD105 was positive, otherwise CD45 and CD34 expression was negative.

In this experiment, CD34, CD45, CD73, CD90, and CD105 were selected as specific surface antigens detected. CD73, CD90, and CD105 were abundantly expressed in all of the 5 generations, while CD34 and CD45 were not. The expression of CD105 in the A5 group is lower than that in the A1-A4 groups (Figure 1).

### 3.2 Cell morphology and expansion capacity of DPSC in different attachment generations

**FIGURE 2:**
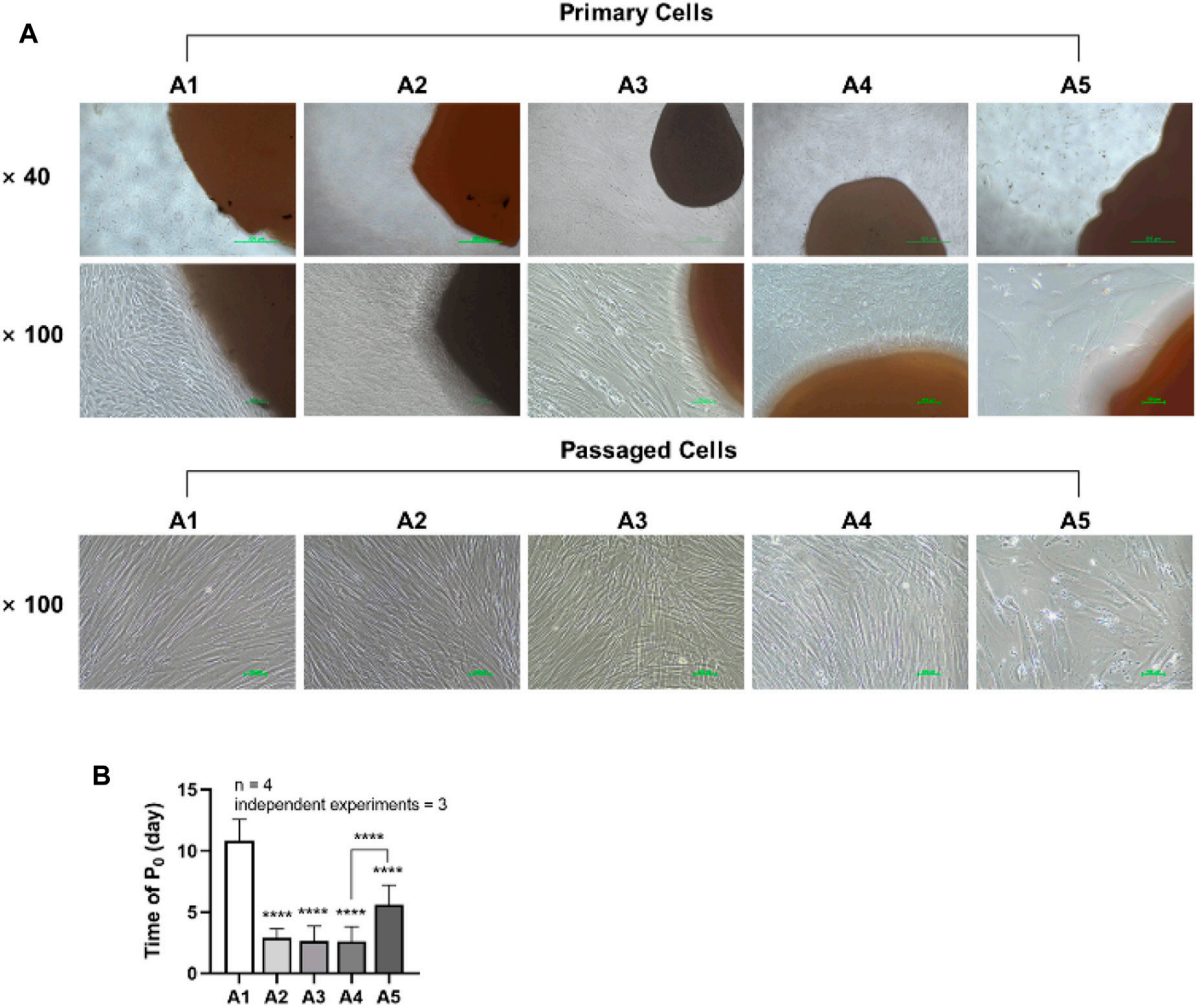
Morphology and emergence time of DPSC in each group. **(A)** Morphology of dental pulp cells in different attachment groups. **(B)** The first appearance time of DPSC in each group. Length of scale bars: 500 μm (×40); 100 μm (×100). ****p < 0.0001 vs. control (A1).

DPSCs were observed daily under a microscope, and the shortest appearance time and each group’s cell morphology were recorded. Two trained experimenters evaluated the cell morphology of each group. Cohen’s kappa coefficient is 86.5% after calculation, which indicates good agreement between the two experimenters. Cells in groups A1-A4 generally exhibited a triangular or spindle shape, with darker cytoplasm and smaller nuclei. In contrast, the cells in group A5 showed more pronounced alterations, displaying striped or irregular morphology, lighter cytoplasm, and significantly larger nuclei (Figure 2A). The shortest appearance time of cells in the A2-A5 group was significantly reduced compared to the A1 group. However, the shortest appearance time of cells in the A5 group rebounded when compared to the A4 group (*p < 0.05*) (Figure 2B).

### 3.3 Effects of new culture strategy on the primary cell yield

**FIGURE 3:**
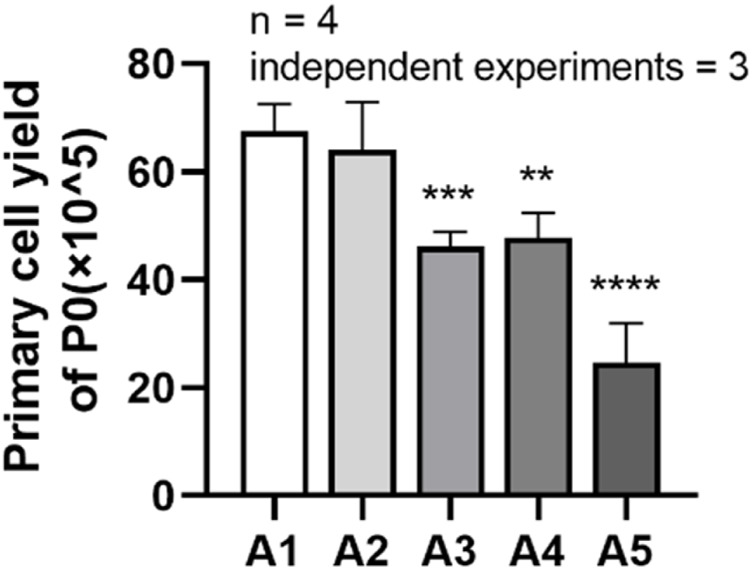
Primary cell yields of DPSC in each group. **p < 0.01; ***p < 0.001; ****p < 0.0001 vs. control (A1).

It can be seen from the figure that the cell yield in group A2 was (64.06 ± 8.792) × 10^5^, while the cell harvest in group A3 decreased to (46.19 ± 2.641) × 10^5^; the total cell yield in group A4 was (47.69 ± 4.638) × 10^5^, while the cell harvest in group A5 decreased to (24.63 ± 7.321) × 10^5^. Compared to the A1 group, the cell harvest in groups A3 to A5 showed a significant downward trend, and the difference was statistically significant. (*p* < 0.05) (Figure 3).

### 3.4 Effects of new culture strategy on the cell survival rate

**FIGURE 4:**
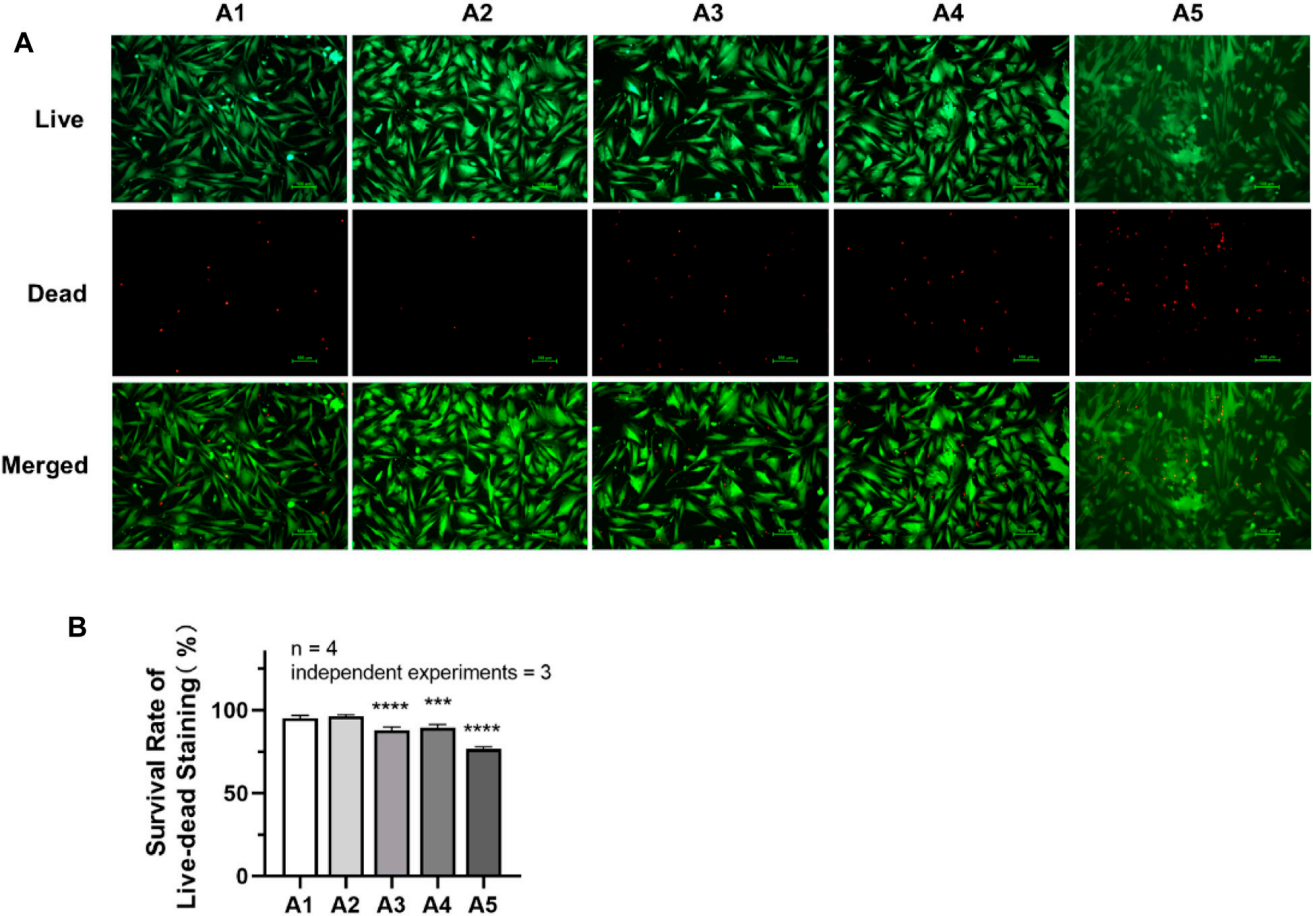
Cell survival rate of DPSC in each group. **(A)** Live–dead staining pictures of DPSC under inverted fluorescence microscope (×100). **(B)** The survival rate of live–dead staining in each group. Length of scale bars: 100 μm (×100). **p < 0.01; ***p < 0.001; ****p < 0.0001 vs. control (A1).

As shown in Figure 4, the cell survival rate of DPSC in the A3-A5 groups was significantly reduced compared with A1 group (*p <* 0.05). The cell survival rate in the A1 group is (95.08 ± 1.687) %. While in A3, A4 and A5 groups, the cell survival rate has decreased to (87.72 ± 2.089) %, (89.31 ± 2.015) % and (76.68 ± 1.305) %, respectively.

### 3.5 Effects of new culture strategy on cell proliferation of DPSC

**FIGURE 5:**
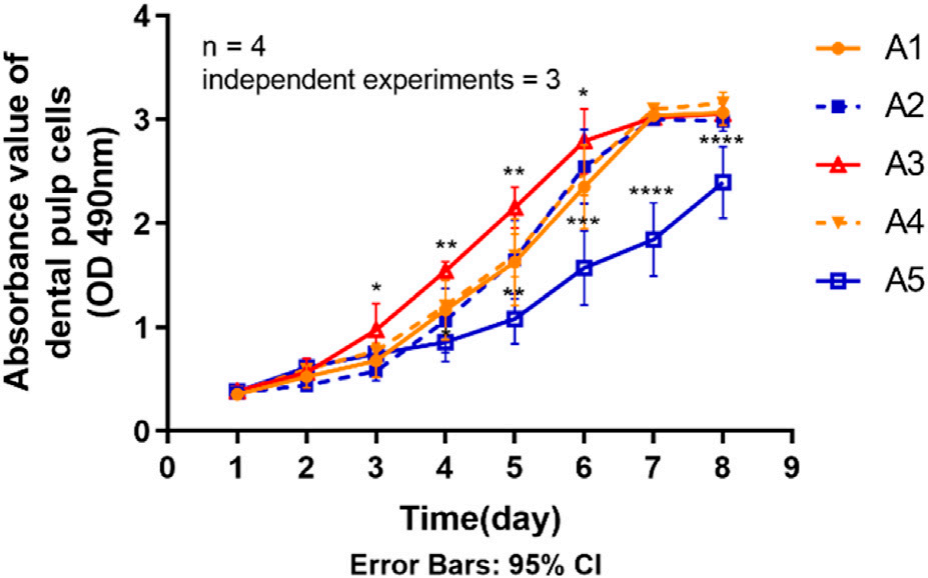
Proliferation ability of primary dental pulp cells in each group. *p < 0.05, **p < 0.01; ***p < 0.001; ****p < 0.0001 vs. control (A1).

The statistical results in Figure 5 show that the proliferative capacity of DPSC was significantly reduced in group A5 compared to the control group (A1). Generally, the rate of increase in the proliferation curve of the cells in group A5 was significantly lower than that of the other groups, as can be seen from the proliferation curves of the 8 days. The absorbance values were significantly lower (*p <* 0.05) in group A5 compared to the control group on days 4–8. However, on days 3–6, the absorbance values of group A3 all exhibited a trend to be higher than the control group (*p <* 0.05). The absorbance values of the other two groups at the same time points were not significantly different (*p >* 0.05) compared to the control group.

### 3.6 Effects of new culture strategy on the osteogenic differentiation potential of dental pulp cells

**FIGURE 6:**
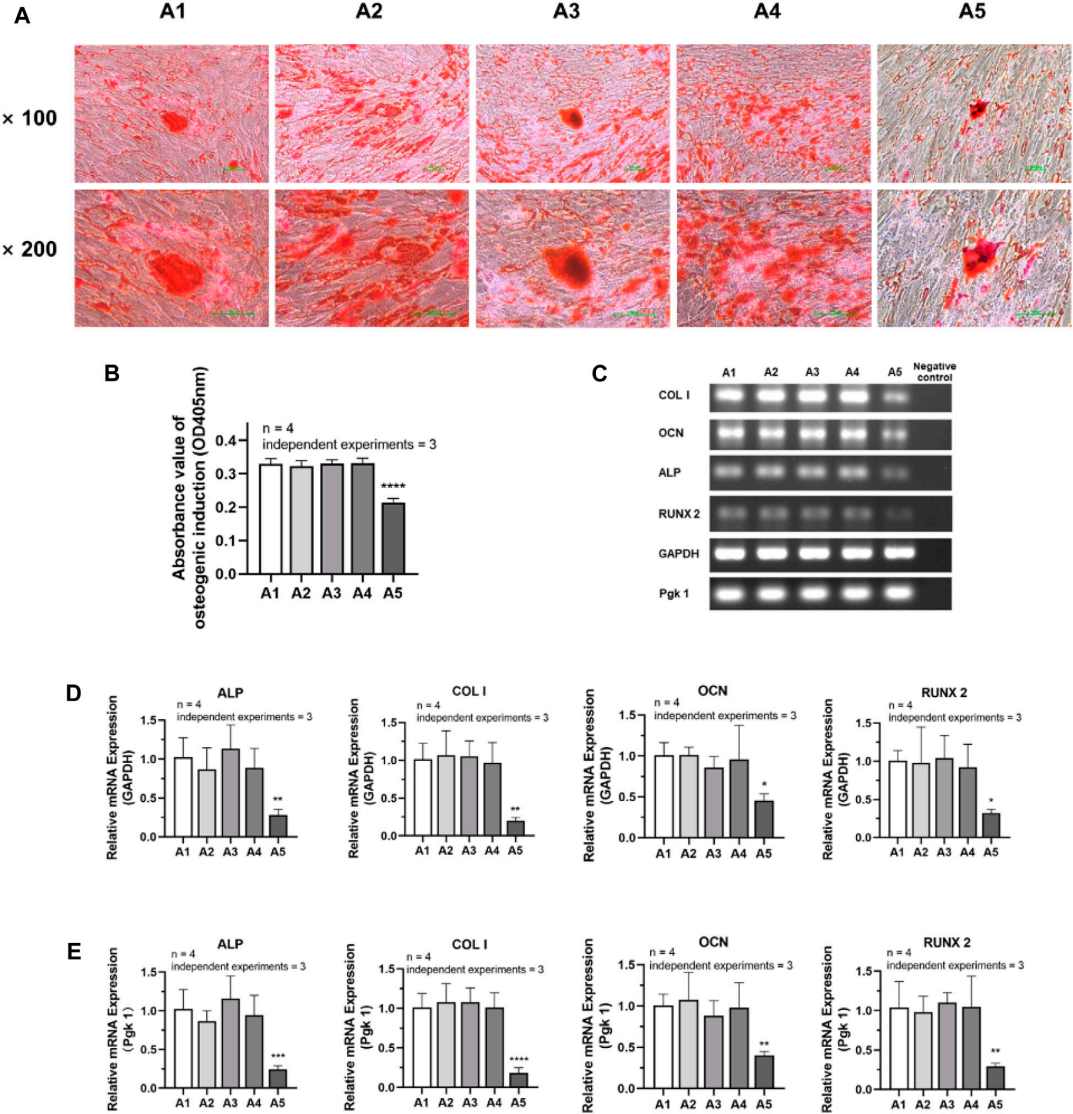
Osteogenic differentiation potential of DPSC in each group. **(A)** Alizarin Red staining after osteogenic differentiation results of DPSC. **(B)** The absorbance value at 450 nm of dissolved solution after Alizarin Red S staining in different groups. **(C)** Gel electrophoresis results of osteogenic gene expression in different groups. **(D)** The relative osteogenic mRNA expression value in different groups by qPCR. GAPDH was selected as reference gene. **(E)** The relative osteogenic mRNA expression value in different groups by qPCR. Pgk 1 was selected as reference gene. Length of scale bars: 100 μm (×100, ×200). ∗*p <* 0.05, ∗∗*p < 0.01*, ***p < 0.001, ****p < 0.0001 vs. control (A1).

The experimental results of osteogenic induction showed a significant decrease in the osteogenic capacity of DPSC in the A5 group. The results of Alizarin red staining showed that the A2-A4 group had densely distributed red-stained calcium nodules similar to the A1 group. However, in group A5, the number of calcium nodules was significantly reduced, resulting in a relative decrease in their density (Figure 6A). Therefore, the absorbance of calcium nodule lysate in group A5 was significantly lower than that in group A1 (Figure 6B) (*p < 0.05*). Detection of the primary osteogenic genes in each group also showed that the expression of osteogenic genes was significantly lower in group A5, including ALP, COL Ⅰ, OCN, and Runx2, with a statistically significant difference (Figures 6C–E) (*p <* 0.05).

### 3.7 Effects of new culture strategy on the adipogenic differentiation potential of dental pulp cells

**FIGURE 7:**
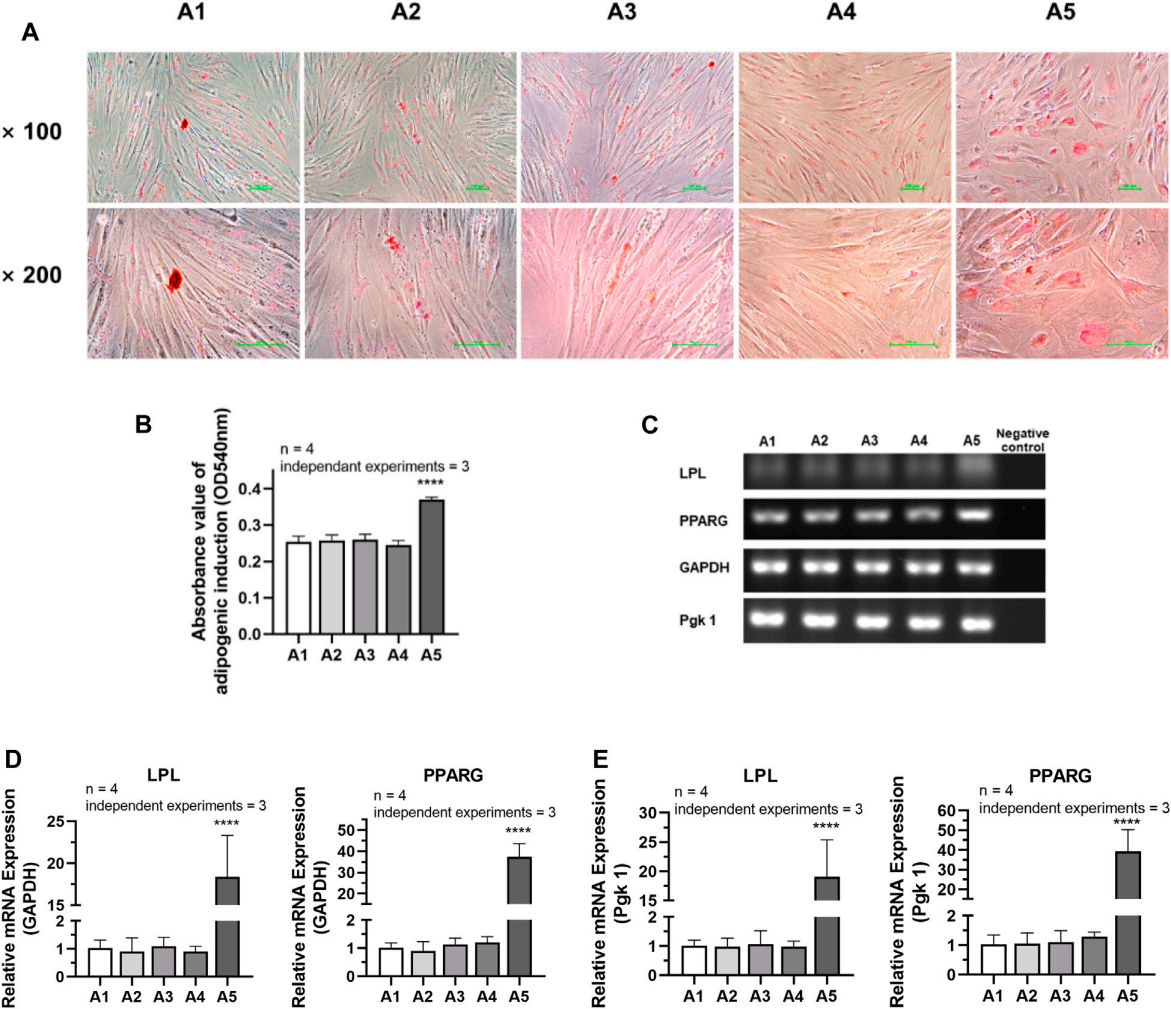
Adipogenic differentiation potential of DPSC in TCH. **(A)** Oil Red O staining after adipogenic differentiation results of DPSC. **(B)** The absorbance value at 540 nm of dissolved solution after Oil Red O staining in different groups. **(C)** Gel electrophoresis results of adipogenic gene expression in different groups. **(D)** The relative adipogenic mRNA expression value in different groups by qPCR. GAPDH was selected as reference gene. **(E)** The relative adipogenic mRNA expression value in different groups by qPCR. Pgk 1 was selected as reference gene. Length of scale bars: 100 μm (×100, ×200). ****p < 0.0001 vs. control (A1).

According to the analysis of the results of adipogenic induction, the adipogenic differentiation ability of DPSC in the A5 group increased remarkably. Lipid droplets around the nucleus were more evident in the A5 group (Figure 7A). Dissolved solution results showed a statistically significant rise in the absorbance of lipid droplet lysate in the A5 group (Figure 7B) (*p < 0.05*). Detection of the related genes in each group also showed that the expression of adipogenic genes was significantly higher in group A5, including LPL and PPARG, with a statistically significant difference (Figures 7C–E) (*p <* 0.05).

## 4 Discussion

In order to validate the feasibility of the new DPSC culture strategy, as well as to enable the collection of as many healthy stem cells as possible, the present experiment was carried out with repeated cultures of dental pulp tissues using the explant method, and the properties of the DPSC obtained in each generation were further examined. According to the guidelines of the International Society for Cell & Gene Therapy (ISCT) (Viswanathan et al., 2019), the corresponding surface antigens of each group of DPSC were detected. In groups A1-A5, CD73, CD90 and CD105 expression was positive, . However, the expression of CD34 and CD45 was negative, which demonstrated that each group of DPSC had the typical features and stemness of MSC (Qu et al., 2020). However, in the A5 group, the expression of CD105 was significantly lower than that in the A1-A4 groups, which may predict the reduced cell proliferation performance of DPSC in the A5 group (Pham et al., 2019). The delay in the earliest appearance of cells in the A5 group and cell proliferation assay results similarly demonstrated a significant decrease in the proliferative capacity of DPSC in the A5 group, especially the cells in the A3 group, which showed higher proliferation activity. Further live-dead cell staining experiments demonstrated that the survival rate of DPSC was also significantly reduced in the A5 group. Furthermore, after 21 days of osteogenic and adipogenic induction of DPSC, we found that the osteogenic capacity of DPSC in group A5 was significantly inhibited, yet their adipogenicity was significantly increased. This was strongly evidenced by the reduced expression of essential osteogenic genes (including ALP, COL Ⅰ, OCN, and Runx2) and the dramatically increased expression of lipogenic genes (LPL and PPARG).

Our group’s experiments demonstrated the feasibility of the optimized cell culture method. Our experimental results confirmed that all DPSC harvested by less than 4 explants had relatively good properties. This optimized approach reduces the cost of cell culture and significantly enhances the amount of healthy DPSC that can be harvested. However, the morphology of DPSC cells obtained by the 5th outgrowth method was markedly changed; not only the cell harvest was greatly diminished, but also the cell viability, cell proliferation ability, and osteogenic ability were decreased in different degrees. On the contrary, their differentiation tendency to adipogenic was markedly improved, which suggested that the cells in the A5 group had already shown a trend of senescence. This is an unfavorable factor for tissue engineering applications. Therefore, DPSCs harvested from group A5 are not recommended for tissue repair. Alraies et al. reported cellular senescence phenomena in dental pulp stem cells (DPSC) after excessive passaging, manifested by the loss of stem cell markers, reduced osteogenic and chondrogenic differentiation, and increased adipogenic differentiation (Alraies et al., 2017). These characteristics are similar to those observed in the A5 group’s harvested cells, suggesting the potential entry of A5 group DPSC into a senescent state. Notably, previous studies have used specific vital proteins as indicators of cellular senescence, with senescence-associated β-galactosidase (SA-βG) activity being a prominent example. However, some argue that the expression of SA-βG lacks specificity, making it unsuitable as the sole parameter for accurate assessment of cellular senescence (Yang and Hu, 2005). Therefore, to comprehensively determine the state of A5 group cells, further evaluation of relevant proteins is necessary to obtain more direct evidence, including P21, P16, phosphorylated-P53, and phosphorylated-RB, is required (Gonzalez-Gualda et al., 2021). This will be further validated in our future experiments.

Furthermore, for accurate discrimination among different cellular states, encompassing cycling cells, stressed cells, quiescent cells, and senescent cells, additional examination of the expressions of Ki67 and pRPS6 is justified (Alessio et al., 2021). Also, careful consideration should be given to selecting reference genes. Some scholars have proposed that commonly used reference genes, such as GAPDH and β-actin, exhibit differential expression across different tissues (Eisenberg and Levanon, 2013). Additionally, experimental findings by McLoughlin K J suggest that the expression of some classical reference genes may be unstable in aging endothelial colony-forming cells (McLoughlin et al., 2019). In a study conducted by Panina Y et al., 2018, 12 reference genes within reprogrammed induced pluripotent stem cells were examined, revealing Pgk1 as one of the most stably expressed reference genes (Panina et al., 2018). Therefore, Pgk1 was rationally and necessarily selected for use in this experiment as an additional reference gene for the corresponding qPCR assay, which can be stably expressed in senescence-prone cells.

Adult-derived mesenchymal stromal cells (MSC) have self-renewal and multipotent differentiation potential, and there are virtually no ethical issues involved with embryonic stem cells, making them regarded as an ideal candidate as a source for regenerative medicine and tissue engineering (Anitua et al., 2018). MSC can have many tissue sources, including but not limited to bone marrow (Mathiasen et al., 2020), adipose tissue (Ong et al., 2021), and placenta (Shin et al., 2021). Although MSC from the above tissue sources has demonstrated good biological properties, most have an obvious disadvantage in stem cell collection compared to dental pulp-derived MSC, i.e., the invasive nature of the manipulation can result in tremendous trauma to the donor site (Ducret et al., 2015; Kang et al., 2016). Conversely, DPSC has more advantages in this regard, including tissue accessibility, lower cost, and less trauma to the donor, as they are mainly derived from third molars (Ledesma-Martinez et al., 2016) and teeth that need to be extracted for orthodontic treatment (Ferrua et al., 2017b), which is often considered as biological waste. In fact, after decades of research, the application of DPSC has entered the animal experimental stage, and most of them have obtained relatively satisfactory results. Mu et al. applied DPSC combined with stem cell factors to repair a rabbit model of facial nerve injury. They found that excellent nerve fiber repair was achieved at 12 weeks postoperatively, similar to the results of the autologous nerve bridging group (Mu et al., 2022). Hu et al. used DPSC intravenously to treat female mice in the Sjögren’s syndrome (SS) model. They found that SS symptoms were significantly reduced, salivary flow was increased, and glandular inflammation was attenuated in the treatment group compared to the control group (Hu et al., 2023). Aliaghaei et al. used DPSC to restore a rat model of cerebellar ataxia. They demonstrated that motor coordination and muscle activity were improved, and inflammatory cell levels were significantly reduced in the animal model (Aliaghaei et al., 2019). Nezhad et al. used DPSC for the treatment of a rat model of mild ischemic stroke (IS). They found that symptoms of IS-induced neurological and tissue deficits were significantly attenuated, the expression of relevant target genes was reduced, and its damaging effects were ameliorated in the short term (Nezhad et al., 2023). In general, because of the excellent proliferative capacity and multipotent differentiation potential of DPSC, they have a wide range of applications in tissue engineering, as confirmed by many experiments.

Numerous studies have investigated the cellular performance of dental DPSC obtained through the explant technique. While most of these studies focused on the initial harvest of DPSCs, they consistently demonstrated the explant technique’s reliability and the harvested cells’ favorable cellular performance. One such study by Spath L et al., in 2010 compared DPSCs obtained via explant technology (hD-DPSC) with those obtained through enzymatic digestion. This study found no significant differences between the two methods in proliferative capacity, osteogenic, and chondrogenic differentiation abilities (Spath et al., 2010). Notably, our study builds upon this by examining cells obtained through the explant method from the 1st to 5th generations. This comprehensive analysis showcases both the advantages and limitations of the explant technique in harvesting DPSCs while ensuring optimal cellular performance.

Vasconcellos Machado C et al. conducted a similar study in 2015, focusing on the first generation of DPSCs obtained through explant technology (de Vasconcellos Machado et al., 2015). Their findings align with our observations, confirming the favorable characteristics of stem cells obtained through the explant method.

Our study introduces a crucial distinction compared to Patil VR et al.'s 2018 publication, which shares a similar methodology involving the repetitive use of explant culture. While both studies employ explant culture for DPSC retrieval, Patil VR et al. did not relocate the tissue block, collecting only enzymatically digested DPSCs that grew out (Patil et al., 2018). Our approach involves relocating the tissue block, facilitating the continuous emergence of subsequent DPSC generations. The results demonstrate excellent proliferative and differentiation capabilities up to the 4th collection but a noticeable decline in cell morphology, proliferative capacity, and osteogenic differentiation ability from the 5th collection onwards. Moreover, there is a significant increase in adipogenic differentiation tendency, highlighting the limitations of DPSC retrieval through explant culture.

Addressing a gap in existing research, La Noce et al. (2014) emphasized the reliance on animal models over clinical studies for *in vivo* applications of DPSCs. They also highlighted the risk of immunological rejection in cell transplantation, emphasizing the importance of biosafety in cell therapy. Our experimental design aligns with these concerns by utilizing explant culture to maximize dental pulp tissue usage, establish personalized stem cell banks, and ensure biosafety by avoiding immunological rejection issues. In conclusion, successfully applying DPSCs in humans requires clinical and fundamental research advancements. Our study provides valuable insights into the limitations and advantages of the explant method, paving the way for future research directions and emphasizing the need for biosafety measures in cell therapy.

Because DPSC has a promising application in various diseases, there may be a significant demand for DPSC for tissue engineering in the future to cope with the therapeutic needs of different diseases. Two widely employed methods for DPSC isolation are the enzymatic digestion of tissues and the explant method. Each method has advantages and disadvantages, as Ferrúa et al. highlight (Ferrua et al., 2017a). Published studies indicate no significant difference in the proliferative capacity, differentiation potential, and expression of antibodies such as CD29, CD44, CD90, and CD105 of DPSCs isolated through these two distinct methods (Hilkens et al., 2013). Enzymatic digestion yields exponentially more cells than the explant method, and the isolation time is shorter. However, the lack of standardization in enzymatic methods for DPSC dissociation may lead to irreversible cell damage, with inappropriate enzymes, excessive concentrations, and prolonged digestion times negatively impacting cell performance (Huang GT. et al., 2006). In contrast, the explant method’s primary advantage lies in its cost-effectiveness, as it does not require digestive enzymes. By affixing as many tissue blocks as possible, DPSC loss during the procedure is minimized, thereby increasing the harvest yield. As mentioned earlier, one potential problem in using DPSC is the small size of the pulp tissue, resulting in a relatively small number of isolated stem cells (Tatullo et al., 2015). It has been suggested that the substantial reduction in pulp tissue due to enhanced secondary dentin formation and root canal mineralization in older individuals has made the isolation of DPSC more challenging (Murray et al., 2002). Preserving DPSC when the donor is relatively young is an effective measure to address this issue, simultaneously ensuring stem cells’ biological properties. Although autologous DPSCs have relatively few ethical concerns and are considered ideal seed cell candidates for tissue engineering without immune rejection, there is no consensus in the academic community on standardized cryopreservation measures. Several methods for pulp cell freezing have been proposed in recent decades. The most common is direct cell cryopreservation after isolation of DPSC from pulp tissue (Huynh et al., 2017; Pilbauerova et al., 2021), which is a well-established and safe method of preservation but has the disadvantage of being time-consuming and costly. Takeshita-Umehara et al. (2023) proposed an interesting new method in which fresh pulp tissue was minced, clamped with cell culture inserts, and cultured for 5 days before freezing the tissue blocks, thus preserving a more significant number of DPSC. Gioventu et al. (2012) also proposed a very constructive method of using a Nd: YAG laser beam to excavate microchannels in the tooth, allowing the cryopreservation agent to contact the pulp and then cryopreserve the entire tooth, which also greatly simplifies the process of DPSC preservation. Dimethyl sulfoxide (DMSO) is the most common cryoprotective agent in most studies, but it has been suggested that even low doses of DMSO may have some cytotoxicity (Galvao et al., 2014). Furthermore, the freezing operation inevitably forms intracellular and extracellular ice crystals, leading to irreversible damage to cell membranes and intracellular structures, further reducing cell survival (Whaley et al., 2021). Although methods to improve cell viability have been continuously proposed, such as controlled rate freezing, rapid freezing (vitrification), and magnetic freezing techniques (Pilbauerova et al., 2019), maximizing cell utilization is also a noteworthy issue for DPSC, a cell with a low tissue source.

To address this issue, our experiments optimized the explant method and explored the maximum number of times the repeated explant method could be used while ensuring the performance of DPSC. The experimental results demonstrated that DPSC obtained with up to 4 times of explant were reliable and had biological properties close to those of primary cells, including cell number, cell proliferation, and osteogenic differentiation ability. Through this study, the cost of cell culture was reduced while the utilization of DPSC was increased, providing a novel idea for applying DPSC in tissue engineering.

## 5 Conclusion

Our current experiment aimed to explore the feasibility of the optimized explant technique and the maximum number of repeated explants while ensuring the biological performance of DPSC. The results of this experiment indicated that DPSC harvested from the 2nd to 4th explants had similar biological properties compared to those obtained from the first explant. Although the survival rate of cells obtained from the 3rd to 4th explants decreased, their proliferative capacity and cell stemness were basically preserved unchanged. Therefore, DPSC obtained from explants with several times up to 4 were reliable. However, starting from the 5th explant, the viability, proliferative, and osteogenic differentiation capacity of DPSC decreased significantly, and the more pronounced morphological changes and adipogenic tendency of the cells indicated that they had entered the senescent state. Our results provide new ideas and references for fully utilizing DPSC in tissue engineering and can provide a more theoretical basis for regenerative medicine.

## Data Availability

The original contributions presented in the study are included in the article/supplementary material, further inquiries can be directed to the corresponding authors.
